# Marine Oil from *C. finmarchicus* Enhances Glucose Homeostasis and Liver Insulin Resistance in Obese Prediabetic Individuals

**DOI:** 10.3390/nu14020396

**Published:** 2022-01-17

**Authors:** Milena Burhop, Jan Philipp Schuchardt, Josefine Nebl, Mattea Müller, Ralf Lichtinghagen, Andreas Hahn

**Affiliations:** 1 Institute of Food Science and Human Nutrition, Leibniz University Hannover, 30167 Hannover, Germany; jan-philipp.schuchardt@lw.uni-hannover.de (J.P.S.); josefine_nebl@web.de (J.N.); mueller@nutrition.uni-hannover.de (M.M.); hahn@nutrition.uni-hannover.de (A.H.); 2 Institute for Clinical Chemistry, Hannover Medical School, 30625 Hannover, Germany; Lichtinghagen.Ralf@mh-hannover.de

**Keywords:** prediabetes, intermediate hyperglycaemia, glucose metabolism, insulin resistance, marine oil, inflammation

## Abstract

The intermediate state between normal glucose tolerance and overt type 2 diabetes mellitus is associated with micro- and macrovascular diseases, requiring safe and cost-effective treatment measures interventions. A novel source of LC n-3 FAs is *Calanus finmarchicus* Oil, which showed promising effects on glucose homeostasis in preclinical studies due to anti-obesity effects and/or anti-inflammatory properties. In total, 43 obese patients (BMI: 31.7 ± 5.2 kg/m^2^) were allocated in the following two groups: (1) Calanus oil group (2 g CO/day) and (2) placebo group (2 g paraffin oil/day). Markers of glucose metabolism, body composition and energy intake were measured at the beginning (t0), after 12 weeks (t_12_) and 16 weeks (t_16_). Overall, parameters reflecting abnormal glucose homeostasis and insulin resistance in the liver, including fasting insulin (−2.9 mU/L ± 4.10, *p* < 0.05), HOMA-IR (−0.9 ± 1.28, *p* < 0.05) and hepatic insulin resistance index (−1.06 ± 1.72 × 10^6^, *p* < 0.05) significantly enhanced after a 12-week CO-intervention, while no differences were observed in HbA1c, AUC_0–2h_ Glucose, AUC_0–2h_ Insulin, 2 h plasma glucose and muscle insulin sensitivity index. Our results indicate that Calanus oil causes beneficial effects on glucose metabolism and insulin resistance in obese patients, with clinical relevance to be verified in further studies. In addition, the possible active compounds and their mechanisms of action should be elucidated.

## 1. Introduction

Due to the aging of the population and the increasing prevalence of various risk factors (e.g., abdominal obesity and metabolic syndrome), the prevalence of type 2 diabetes mellitus (T2DM) has risen rapidly worldwide in recent decades. T2DM is a chronic metabolic disorder with hyperglycaemia resulting from the disturbance of insulin action on peripheral tissues such as the liver and muscle and/or pancreatic insulin secretion [1]. The pathogenesis of T2DM is multifactorial, but abdominal obesity and associated dysfunctions of the visceral adipose tissue (VAT) (e.g., increased secretion of non-esterified fatty acids, hormones and pro-inflammatory mediators) seem to play a key role in developing insulin resistance [2]. With an annual conversion rate of 5–10% and a lifetime risk of about 70%, prediabetes is a phase between normal glucose tolerance (NGT) and overt T2DM. Prediabetes can be classified into two abnormalities with different pathologies: impaired fasting glucose (IFG) and impaired glucose tolerance (IGT) based on the 2 h glucose tolerance test (OGTT) or a combination of both [3,4,5]. IFG and IGT are associated with the predicted conversion rate to T2DM. Annualized incidence rates for isolated IGT (4–6%) and isolated IFG (6–9%) are lower than both IFG and IGT (15–19%) [6], indicating that combined IFG and IGT are an advanced disease state. According to recent clinical studies, prediabetes is not only a well-established risk factor for the progression to T2DM, but it is also associated with micro- and macrovascular diseases such as coronary heart disease (CHD) and diastolic heart failure, even without manifested T2DM [7].

In recent decades, long-chain polyunsaturated fatty acids (LC n-3 FAs), namely docosahexaenoic acid (DHA, 22:6n-3) and eicosapentaenoic acid (EPA, 20:5n-3) have received growing attention due to their protective and health-promoting effects, primarily on cardiovascular health [8,9,10]. LC n-3 FAs are attributed with numerous beneficial properties, including the reduction in inflammatory conditions, contributing to preventive and treatment effects for cardiovascular diseases and vascular inflammation, among others, as shown in manifold observational studies [11,12]. Besides reducing the risk of cardiovascular diseases, LC n-3 FAs have been shown to be associated with a lower incidence of diabetes [13]. In brief, the presumed beneficial health effects of LC n-3 FA in insulin resistance (IR) and T2DM include the following: (1) down-regulating of nuclear factor kappa B (NFκB) pathways via GPR120, resulting in reduced low-grade inflammation; (2) generation of pro-resolving mediators; (3) up-regulating of AMP-activated protein kinase (AMPK) [14]. However, the evidence from randomized controlled trials remains contradictory [15].

A novel source of LC n-3 FAs is the marine Calanus oil (CO), extracted from *Calanus finmarchicus*, a copepod predominantly found in the North Atlantic Ocean. Due to the excessive abundance of *C. finmarchicus*, CO provides a resource-saving and sustainable alternative to oils from commercial fish stocks [16,17]. While LC n-3 FAs in marine mammals as fish or Antarctic krill are primarily bound as triglycerides (TAG) or phospholipids (PL), respectively, the bonding form of LC n-3 FAs in CO differs fundamentally from conventional oils. Of all the FAs contained in CO, more than 80% are bound as wax esters (i.e., FA that are esterified with unsaturated fatty alcohols (FAOH)) serving as energy stores for crustaceans such as *C. finmarchicus* [18]. Besides well-known LC n-3 FAs such as DHA, EPA and their precursor stearidonic acid (SDA, 18:4n-3), the FA fraction contains monounsaturated fatty acids (MUFA) such as gondoic acid (20:1n-9) and cetoleic acid (22:1n-11). In addition, CO contains smaller amounts of plant sterols and carotenoids, particularly astaxanthin. Astaxanthin is one of the most potent antioxidants found in nature and, similarly to LC n-3 FAs, has recently been associated with health-promoting effects in obesity-induced metabolic disorders [19,20,21].

Previous preclinical studies with mice showed promising effects of CO on glucose homeostasis, mainly resulting from anti-obese effects and/or anti-inflammatory properties [22]. However, no studies have been carried out investigating the clinical efficacy of CO on glucose homeostasis in obese or diabetic patients. Thus, in this proof-of-principle study, we aimed to confirm the effects of CO on glucose metabolism and insulin resistance in obese patients. To exert beneficial health effects, the bioavailability of LC n-3 FAs is considered a crucial precondition. Therefore, the potential of CO-derived wax esters to increase LC n-3 FAs supply status was determined using the Omega-3 Index (O3I).

## 2. Materials and Methods

### 2.1. Study Design and Participants

The randomized, placebo-controlled and double-blinded study was conducted at the Institute of Food Science and Human Nutrition in Hannover, Germany. Previously, the Ethics Committee of the Medical Chamber of Lower Saxony (Hannover, Germany) provided ethical approval for the realization of this research work. In accordance with the Guidelines of the Declaration of Helsinki, written informed consent was obtained from all participants prior to their enrolment. This study is officially recorded in the German Clinical Trials Register (DRKS00017537).

Briefly, the study consisted of a screening phase and a 16-week intervention period with three examinations at baseline (t_0_), after 12 weeks (t_12_), and at the end of the intervention (t_16_). Main inclusion criteria for participation were: FPG > 100–125 mg/dL (5.7–6.9 mmol/L; ADA criteria for fasting glucose); age ≥ 40 and ≤75 years; a stable body weight within the last six months (±3 kg); an omnivorous diet; a fish consumption of <1 portion/week and disordered glucose homeostasis. Exclusion criteria were defined as follows: suffered with chronic diseases (i.e., T2DM, cardiovascular diseases, and cancer), especially chronic diseases involving the immune system (i.e., Crohn’s diseases, colitis ulcerosa, rheumatoid arthritis, and multiple sclerosis); medication or supplements, affecting blood glucose control and insulin sensitivity (i.e., isolated dietary fibre such as guar gum, pectins or psyllium); regularly consumption of fatty fish/week; use of immunosuppressive drugs (i.e., corticoids), alcohol, drug and/or medicine dependency; pregnancy or lactation; concurrent participation in another clinical study, and participation in a trial in the last 30 d.

The participants were recruited via press releases and advertisements in local newspapers, medical practices as well as pharmacies of Hannover and the surrounding countries. For participant recruitment, the Institute for Food Science and Human Nutrition in Hannover was also cooperating with general practitioners. The participants were randomly assigned by an independent researcher using stratified randomisation according to the covariates (in descending order: waist circumference, sex, age) to one of the following two groups: control/placebo group (CON) and Calanus oil supplementation group (CO group).

Over the 16-week intervention period, the CO group received LC n-3 FAs rich oil extracted from *C. finmarchicus* (Calanus AS, Tromsø, Norway); while control group was supplemented by placebo capsules (filled with paraffin oil). The participants were advised to consume four capsules daily (2 g CO/day) with plenty of water (at least 200 mL) and a meal to ensure LC n-3 FAs absorption. Tande et al. (2016) evaluated the clinical safety of CO (2 g/d) using biochemical and haematological parameters in a randomized, double-blind, placebo-controlled trial. In summary, no adverse effects on human body were observed [23]. Additionally, in the present trial, tolerability was controlled using questionnaires. To assess compliance, participants received a predefined number of capsules (>85% had to be consumed), which was counted at the end of the intervention. The composition of Calanus oil is shown in Table 1.

### 2.2. Monitoring of Dietary Intake and Physical Activity

The dietary intake of the participants was documented before intervention (t_0_), after 12 weeks (t_12_) and at the end of intervention (t_16_) via 3 d dietary records including two working days and one weekend day. To increase the internal validity, the records were checked for plausibility; if necessary, ambiguities were clarified directly with the participants. Energy and nutrient intake were estimated by using PRODI6.4^®^ software (Nutri-Science GmbH, Freiburg, Germany).

The physical activity was recorded and controlled using a questionnaire (includes frequency and type of activities) at time points t_0_, t_12_ and t_16_. Overall, the participants were instructed not to modify their dietary habits (especially in view of the intake of LC n-3 FA rich foods) or physical activities during the intervention period to minimize nutritional effects on variability in LC-PUFA status and glucose metabolism.

### 2.3. Anthropometric Measurements and Body Composition

Body height was measured using a stadiometer (Seca GmbH & Co. KG, Hamburg, Germany); participants were instructed to touch the stadiometer with the back of the head, the back, the buttocks and the back of the feet. Body weight was measured digitally (Seca GmbH & Co. KG, Hamburg, Germany) to the nearest of 0.1 kg (lightly clothed, without shoes). Body composition was evaluated using a bipolar bioelectrical impedance analyser (BIA) (Nutriguard M, Data Input Company, Darmstadt, Germany) and NutriPlus 5.4.1 software (Data Input Company, Darmstadt, Germany). For the measurements, participants were instructed to lie down on a stretcher and rest for about 5 min to ensure a balanced distribution of body fluids. The participants were instructed to position their limbs bent away from the trunk; they were asked to lie still and relaxed without speaking. To avoid bias due to changing persons, measurements were preferably carried out by the same nutritionists.

### 2.4. Blood Collection and Analysis of Glucose Metabolism Markers

To determine glucose, insulin and HbA1c, blood was drawn from the participants after an overnight fast (≥12 h) between 06.00 and 10.00 h (for each participant at the same time of each examination visit, if possible) by venepuncture of an arm vein using serum and Gluco Exact tubes (Sarstedt AG & Co. KG, Nümbrecht, Germany). These were stored at 5 °C and transferred to an accredited and certified laboratory (Medizinische Hochschule Hannover, Hannover, Germany).

At each examination visit (screening, t_0_, t_12_ and t_16_), a 75 g fasting oral glucose tolerance test (OGTT) was performed (75 g/200 mL, LemonGluc). Blood was drawn at baseline and after 30, 60, 90 and 120 min to measure glucose and insulin concentrations to calculate the area under the curve (AUC) Glucose/Insulin using the trapezoidal method. The participants were advised to have an adequate intake of carbohydrates (i.e., at least 150 g/day) prior to the OGTT. Fasting glucose was determined by a photometric method (Beckman Coulter GmbH, Krefeld, Germany). HbA1c was analysed using high-pressure liquid chromatography (HPLC) (Bio-Rad Laboratories GmbH, Feldkirchen, Germany). The electrochemiluminescence immunoassay method (ECLIA) using cobas 801e (Roche Diagnostics GmbH, Mannheim, Germany) was applied to determine insulin concentrations. To evaluate insulin resistance and beta-cell function, the homeostatic model assessment (HOMA) was calculated based on the method of Matthews et al. (1985) as follows: HOMA-Index = fasting insulin (µU/mL) × fasting blood glucose (mg/dL)/405 [24]. Hepatic insulin resistance index (HIRI) and muscle insulin sensitivity index (MISI) were derived from the 5-point oral glucose tolerance test (OGTT) using the method of Abdul-Ghani et al. (2007) [25]. HIRI was calculated from the product of the area under the curves (AUC) for glucose and insulin during the first 30 min. of the OGTT, while MISI was calculated from the decline in peak plasma glucose concentration (i.e., after around 60–120 min) during the OGTT in relation to plasma insulin concentration as follows:

$$\mathrm{HIRI}=\mathrm{AUC}\;0–30{\;\mathrm{Glucose}\;[\mathrm{in}\;\mathrm{mmol}/\min}^{-1}{/L]\times \mathrm{AUC}\;0–30\;\mathrm{Insulin}\;[\mathrm{in}\;\mathrm{mU}/\min}^{-1}/\mathrm{mL}]$$

MISI=dGlucose/dt÷Plasma Insulin [mean during OGTT mU/L]

### 2.5. Analysis of Omega-3 Fatty Acid Content of Total Fatty Acids in Red Blood Cells

For FA and O3I analysis in red blood cells (RBC), the blood samples (9 mL ETDA tubes, Sarstedt AG & Co. KG) were centrifuged at 3000 rpm for 10 min; the buffy coat was removed and erythrocytes were immediately frozen (at −80 °C). The analysis was performed by the laboratory Omegametrix GmbH (82152 Martinsried, Germany) according to the HS-n-3-Index Methodology^®^ by using gas chromatography [26]. According to the standardized method, fatty acid methyl esters were extracted from the erythrocytes by transesterification; analysed by gas chromatography using a GC2010 gas chromatograph (Shimadzu, Duisburg, Germany) equipped with a SP2560, 100 m column (Supelco, Bellefonte, PA, USA); and subsequently identified using a characteristic standard mixture. Hydrogen was used as the carrier gas. Applying a correction factor, the O3I was determined as the percentage of EPA + DHA in the total FAs content. The quality was ensured according to DIN ISO 15189.

### 2.6. Statistical Analysis

To detect statistically significant between-group differences with a two-sided significance level of 5% (α = 0.05) and a power of 80% (β = 0.8), a sample size of n = 25 per intervention arm is needed. With an expected drop-out rate of 15%, at least 30 participants per group had to be recruited.

Data were presented as mean ± standard deviation (SD). Distribution of all data was assessed using the Shapiro–Wilk test and Gaussian distribution. To detect differences between the groups at baseline, the *t*-test was used for normally distributed data and the Kruskal–Wallis test for non-normally distributed data; for nominal data, the Chi-square test was applied. The intervention effect was determined using a two-way analysis of variance with repeated measures (ANOVA) with time (t_0_, t_12_, t_16_) and intervention (CO, CON). If the data did not meet the prerequisite (i.e., were non-normally distributed), data were transformed using log or square root transformation. In case of significant effects in ANOVA, a post hoc test with Bonferroni correction was performed. At minimum, *p*-values of <0.05 were considered as significant. All statistical analyses were carried out using SPSS software (version 23.0; SPSS Inc., Chicago, IL, USA).

## 3. Results

### 3.1. Baseline Characteristics

After eligibility testing, participants were randomized to one of the two intervention groups, i.e., the Calanus oil group or the placebo/control group. In total, 43 individuals with an isolated IFG and compliance >85% (i.e., 85% of capsules taken) were included; participants with compliance <85% were classified as dropouts (n = 4). The study population (72% female, 28% male) is characterized as obese (Class 1: BMI from 30 to <35) with a mean BMI of 32 and marginally hypercholesterolaemic (total cholesterol > 200 mg/L) with mean total cholesterol levels of 205 mg/dL (5.3 mmol/L).

Baseline characteristics of participants have been summarized in Table 2. No differences in anthropometric and/or biochemical parameters between both groups were observed. Likewise, no significant differences in body composition markers were detected among the intervention groups (Table A1).

### 3.2. Markers of Glucose Metabolism and Inflammation

All at baseline, no significant differences between the two study groups were observed. After 12 weeks of intervention, significant differences in several glucose parameters, including fasting plasma insulin (FPI; *p* = 0.014), HOMA-IR (*p* = 0.028) and HIRI (*p* = 0.021) were observed among the groups. In the CO group, a reduction in FPI (−2.9 mU/L ± 4.10, *p* < 0.05); HOMA-IR (−0.9 ± 1.28, *p* < 0.05) and HIRI (−1.06 ± 1.72 × 106, *p* < 0.05) was found, while the placebo group showed no significant changes (Table 3). However, FPI, HOMA-IR and HIRI increased in the last four weeks but were still below the corresponding baseline value. There were no changes in other parameters of glucose metabolism, including HbA1c, AUC0–2h Glucose/Insulin, 2 h plasma glucose and MISI.

FPG levels decreased in the CO group from 6.1 ± 0.30 mmol/L at baseline to 5.9 ± 0.47 mmol/L after 12 weeks of intervention and remained stable at 5.9 ± 0.43 mmol/L after the 16 weeks of intervention, with no effects observed among the study groups (i.e., time * intervention effect). Overall, 6 of 25 participants reached an FPG < 5.7 mmol/L after a 16-week intervention with CO. In contrast, no significant changes were found in placebo group after intervention, neither from baseline to t_12_ nor to t_16_ (*p* > 0.05).

To assess inflammatory status, C-reactive protein (CRP) was used. Overall, there was a reduction in both groups, i.e., CO group and placebo group from baseline to t_12_, which was not significant among the groups from baseline to t_12_ or to t_16_ (*p* = 0.728, *p* = 0.478).

### 3.3. Omega-3 Fatty Acid Content of Red Blood Cells

At baseline, no differences in several LC n-3 FAs content in RBCs, including ALA, SDA, EPA, DHA and O3I, between both groups were detected (Table 4). The O3I in the CO group after 12- and 16-week intervention increased significantly (*p* < 0.001) from 6.45 ± 1.37% (t_0_) to 7.71 ± 0.54% (t_12_) and 7.72 ± 1.43% (t_16_), respectively. In line, EPA increased from 0.99 ± 0.33% (t_0_) to 1.40 ± 0.44% and 1.39 ± 0.40% (t_16_), respectively, a relative increase of 40% after a 16-week intervention. DHA increased by 20% after a 16-week intervention, i.e., from 5.46 ± 1.14% (t_0_) to 6.31 ± 1.24% (t_12_) and 6.34 ± 1.17% (t_16_), respectively. Further, there was an increase in SDA from 0.047 ± 0.054 (t_0_) to 0.054 ± 0.013% (t_12_) and 0.054 ± 0.011% (t_16_), respectively. However, the effect was not observed between the groups, but within the CO group. Overall, no significant changes in all LC n-3 FAs were detected in the last four weeks.

Noticeably, the O3I significantly decreased after 12 and 16 weeks of intervention in placebo group, i.e., from 7.73 ± 1.96 (t0) to 6.89 ± 1.78 (t12) and 6.94 ± 1.87 (t16), respectively. Otherwise, no significant changes were observed in placebo group, either from baseline at t_12_ or t_16_.

### 3.4. Dietary Intake and Physical Activity

Dietary intake from 3 d dietary records is shown in Table A2. Overall, no significant changes were observed; in particular, there were no changes in nutrients that are beneficial for glucose homeostasis (e.g., fibre) or that affect O3I (e.g., EPA and DHA via consuming LC n-3 FAs rich foods such as cold-water fish).

As expected for the study population, physical activity did not change significantly. Therefore, exercise-induced beneficial effects on glucose metabolism can be ruled out.

## 4. Discussion

Due to the pandemic proportions of T2DM, there is a demand for safe, cost-effective, and long-term interventions to reverse back to NGT [27]. Currently, intensive lifestyle interventions, i.e., the combination of diet and exercise programs and/or pharmacotherapies (e.g., metformin and pioglitazone), are the most effective measures to halt the progression of T2DM [28]. A crucial barrier for elaborate lifestyle modification is patient compliance, resulting in insufficient interventions to permanently reduce the prevalence of T2DM. Therefore, alternative or additional interventions are coming into focus to prevent the manifestation of T2DM in the future.

Abdominal obesity and its metabolic comorbidities, such as chronic low-grade inflammation, appear to be crucial drivers in the pathogenesis of T2DM. VAT acts as an endocrine tissue producing pro-inflammatory mediators (e.g., Il-1ß, IL-6, TNF-α), which interfere with the insulin-signalling pathway locally or systemically in other insulin-sensitive tissues [29]. In contrast, adiponectin levels are reduced in abdominal obesity. Normally, it acts as an insulin sensitizer, stimulating fat and glucose metabolism via AMP-activated protein kinase (AMPK) [30]. Insufficient insulin action also affects fat metabolism, leading to excessive non-esterified FAs release and accumulation of TAG and intermediates (e.g., DAG, Ceramides) in peripheral tissues such as the liver, skeletal muscle, and pancreas. These intermediates activate protein kinase C isoforms that inhibit the insulin-signalling pathway, resulting in whole-body insulin resistance and the manifestation of T2DM [31].

Until now, the effects of CO on glucose homeostasis and insulin resistance in metabolic disorders are limited to preclinical studies with mice, which indicated that glucose tolerance increased by 16%, presumably due to anti-obese and anti-inflammatory properties. VAT and fat accumulation in the liver (i.e., steatosis) were reduced by 27 and 41%, respectively. In addition, adiponectin expression was significantly upregulated, which reduced fat accumulation probably via an AMPK-mediated mechanism. Contrary, wax esters in VAT decreased the expression of pro-inflammatory genes, leading to a more than 70% reduction in macrophage infiltration [22].

In the present proof-of-principle study, a 12-week intervention with 2 g CO reduced FPI, HOMA-IR and HIRI. In the fasting state, 80–85% of plasma glucose is produced in the liver (gluconeogenesis), enabling indices derived from FPG and FPI such as HOMA-IR to assess liver insulin resistance. After a glucose load in the postprandial phase, the rise in plasma glucose stimulates insulin secretion from the pancreatic beta-cells, leading to the suppression of gluconeogenesis. Contrary, glucose production in insulin-resistant individuals is only mildly to moderately suppressed in the early phase of OGTT (0–30 min), increasing glucose and insulin concentrations proportional to the extent of insulin resistance in the liver. HIRI (AUC 0–30 Glucose (mmol/min^−1^/L) × AUC 0–30 Insulin (mU/min^−1^/mL)) considers both basal glucose production and gluconeogenesis suppression; thus, HIRI enables the determination of liver insulin resistance 60 to 120 min. after glucose load of the OGTT, the gluconeogenesis rate remains at a constant level and post load plasma glucose concentration stimulates glucose utilization in peripheral tissues, in particular skeletal muscles. Hence, the decline in plasma glucose concentration after 60–120 min of the OGTT reflects the skeletal muscle insulin sensitivity [25]. Summarized, our results indicate that the insulin-sensitizing effects of CO are organ-specific to the liver.

In contrast to our results, a 4-month intervention RTC with CO providing 230 mg EPA + DHA daily, combined with an exercise program, neither affected insulin nor HOMA-IR in elderly patients (62–80 years, BMI 19.3–37.0 kg/m^2^), but increased glucose utilization in muscle (measured as MFFM (glucose utilization related to fat-free mass)), which is an indicator for insulin-sensitizing effects in muscle [32]. However, evidence of exercise-mediated activation of AMP-kinase, which has been shown to affect glucose metabolism insulin independently [33], contributing to the elevated utilization rate in muscle even without additional CO intervention.

In the present study, patients with isolated IFG were included, showing increased glucose production in the fasting state (FPG of 5.7–6.9 mmol/L) to an excessive rate of liver gluconeogenesis. Individuals with isolated IFG show a NGT in the postprandial state, allowing only the assessment of liver insulin resistance [25,34]. Further studies with patients exhibiting isolated IGT or both, IFG and IGT (2 h plasma glucose > 7.8 mmol/L), are needed to evaluate the beneficial effects of CO on whole-body insulin resistance. However, an intervention time of 16 weeks is comparatively long to ensure unaltered participants’ adherence to the measures. Hence, the lack of beneficial effects in the last 4 weeks could be due to insufficient supplement intake.

Chronic low-grade inflammation and modifications in adipokine patterns are crucial factors in the pathogenesis of insulin resistance and T2DM in obese individuals [35,36,37,38]. Anti-inflammatory properties of LC n-3 FAs are well known [14]. LC n-3 FAs are precursors for eicosanoids and pro-resolving mediators called resolvins, protectins, and maresins, which are known to control and combat chronic inflammation [39]. In recent RCTs, anti-inflammatory effects were found with pure EPA + DHA or high-dose (usually enriched) fish oil interventions (e.g., >2 g fish oil per day, containing 600 mg EPA; 400 mg DHA) [40,41,42]. Regarding low doses in CO, another possible mechanism of action is plausible, as follows: in contrast to fish oil TG-bound LC n-3 FAs, wax esters-derived LC n-3 FAs from CO reach the distal intestine, allowing interactions with abundant G-Protein coupled Receptors (GPR120) in intestinal cells [43]. GPR120 received attention to combat inflammation due to down-regulating NFκB- and JNK-signalling pathways [44]. SDA—the most abundant LC n-3 FA in CO—has a particular role, as it is one of the most potent activators of GPR120. However, Čížková et al., 2020 demonstrated that CO (230 mg EPA + DHA/day) combined with exercise had no additional benefit in attenuating inflammation in elderly patients [32]. In the existing collective of obese patients, at least the mean CRP level at baseline (CO group: 2.81 mg/L; placebo group: 3.88 mg/L) showed no to mild low-grade inflammation (CRP > 3 mg/L) [45]. However, this condition does not exclude the potential of a local inflammation (and CO-induced anti-inflammatory effects). Based on the current evidence from clinical trials, the link between CO and chronic inflammation remains unclear.

It is also inconclusive and unclear whether potential anti-inflammatory effects of CO are directly mediated via LC n-3 FAs or indirectly via their anti-obese effects. In general, LC n-3 FAs exert anti-obese effects via AMPK-mediated stimulation of fat metabolism (i.e., suppressing lipogenic genes; activating genes that encode mitochondrial and peroxisomal beta-oxidation in both the liver and muscle) As a result, VAT and fat accumulation (and intermediate metabolites) in peripheral tissues are reduced [14]. In a recent study, it was showed that moderate exercise combined with 2 g CO per day (200 mg EPA + DHA/day) has a beneficial effect on reducing fat mass [46]. In line with this, Daďová et al. (2020) found a reduction in VAT in obese women after an exercise intervention combined with 2.5 g CO per day (230 mg EPA + DHA/day) [47]. However, we could not observe effects on anthropometric and/or body composition.

In general, to exert beneficial effects, the bioavailability of LC n-3 FAs is considered a critical precondition. Our results demonstrate that the CO supplementation increased the O3I, suggesting that wax ester-bound LC n-3 FAs are well absorbed and long-term available to exert an insulin-sensitizing effect in obese patients with IFG. Of note, the basal LC n-3 FA status of the study population is relatively well at 6.45% O3I (CO group) and 7.37% O3I (CON group) for study participants reporting low fish consumption (<one serving fish per week). In comparable study groups, the mean O3I values were significantly lower at 5.49% [48] or 5.12% [49]. To clarify whether the insulin-sensitizing effects of CO would possibly be even more substantial with a lower LC n-3 FAs status (i.e., O3I < 5%), future studies should recruit participants via pre-screening for a low LC n-3 FAs supply status.

Considering the low doses of CO-derived LC n-3 FAs, several other bioactive compounds (e.g., astaxanthin or plant sterols) present in the oil probably contribute to effects on glucose homeostasis. Preclinical studies with mice showed beneficial effects of astaxanthin in counteracting obesity and obesity-induced comorbidities, particularly inflammation and insulin resistance [19]. However, evidence from clinical studies is limited [50,51]. Moreover, a recent RCT examined the role of a 12-week intervention of plant sterols (PS) (1.7 g/day) and LC n-3 FAs (1000 mg/day EPA, 400 mg/day DHA) both isolated and in combination in individuals with IFG. The results indicate that this combined treatment achieved more beneficial effects on glucose metabolism and inflammation than an isolated treatment (e.g., FPG reduced about five times as much as an isolated intervention with LC n-3 FAs) [52]. However, in the present study, the daily PS dose (7 mg/day) was also considerably lower. Nonetheless, this does not rule out synergistic effects and, rather, it confirms our theories that insulin-sensitizing effects are due to several CO-derived bioactives.

Limitations

Unfortunately, compliance was only assessed at the end of the study (16 weeks) instead of examining the compliance after 12 and 16 weeks. Hence, the increase in FPI, HIRI levels and HOMA index after 12 weeks can probably be ascribed to a decline in supplement intake. Another minor methodological limitation is using 3 d dietary protocols, which can only represent the habitual diet for a limited period. However, the participants were instructed not to change their usual diet during the intervention period. In addition, a change in dietary habits over the short period of 4 months is not to be expected in a collective of elderly patients.

## 5. Conclusions

In conclusion, we demonstrate that CO causes beneficial effects on glucose metabolism and insulin resistance in obese patients with IFG. The clinical relevance of these effects should be verified in future studies. To further elucidate the preventive and treatment potential of CO on insulin resistance, RCTs should be carried out with patients exhibiting isolated IGT or both IFG and IGT. It also seems plausible to pre-screen patients with a low O3I (<5%) and/or systemic low-grade inflammation. In addition, further studies should consider the potential mechanisms of action of CO on glucose metabolism and insulin resistance, i.e., with particular attention on the mediators regulating low-grade inflammation and body composition.

## Figures and Tables

**Table 1:** Composition of Oil extracted form *Calanus finmarchicus*.

Components		g/100 g CO	mg/2 g CO
MUFA		9.7	194
PUFA		26.2	524
	Omega-3 fatty acids	25.0	500
	ALA	1.4	28
	SDA	8.4	168
	EPA	6.9	138
	DHA	6.4	128
	Omega-6 fatty acids	1.1	22
	LA	0.7	14
	ARA	0.2	4
Fatty alcohols		28.8	576
Sterols		0.35	7
Astaxanthin		0.1	2

CO, Oil from *Calanus finmarchicus*; MUFA, Monounsaturated fatty acids; PUFA, Polyunsaturated fatty acids; ALA, Alpha-Linolenic acid; SDA, Stearidonic acid; EPA Eicosapentaenoic acid; DHA, Docosahexaenoic acid; LA, Linoleic acid; ARA, Arachidonic acid.

**Table 2:** Sex, age and anthropometric parameters of the study population at baseline.

Parameter	CO (n = 25) Calanus Oil		*p* (n = 18) Placebo Group		*p* *
	Mean	SD	Mean	SD	
Sex (f/m)	18/7	-	13/5	-	0.98
Age (years)	59.56	9.51	61.78	7.61	0.42
Height (cm)	173.1	10.00	170.2	8.24	0.32
Body weight (kg)	92.98	19.61	94.9	19.14	0.94
BMI (kg/m^2^)	30.95	5.21	32.64	5.27	0.58
Waist circumference (cm)	101.52	15.51	104.67	12.92	0.87
Hip circumference (cm)	115.20	9.83	116.11	9.52	0.76
WHR	0.88	0.10	0.90	0.08	0.41
SBP (mmHg)	136.32	16.42	134.56	15.13	0.72
DBP (mmHg)	83.04	9.96	83.56	8.47	0.86
Pulse (bpm)	68.16	11.76	62.83	6.59	0.09
Total cholesterol (mmol/L)	5.48	1.14	5.18	0.91	0.37
HDL-cholesterol (mmol/L)	1.49	0.44	1.36	0.34	0.28
LDL-cholesterol (mmol/L)	3.74	1.00	3.52	0.90	0.48
TAG (mmol/L)	1.52	0.66	1.38	0.77	0.52

CO, Oil extracted from *Calanus finmarchicus*; f, female; m, male; WHR, waist-hip-ratio; SBP, systolic blood pressure; DBP, diastolic blood pressure; TAG, triglycerides. * Distribution of sexes between groups was analysed using the χ^2^ test. Otherwise, group differences were assessed with the Mann–Whitney U test or unpaired *t* test.

**Table 3:** Effects of Calanus oil on markers of glucose metabolism and inflammation.

Parameter	t	CO (n = 25) Calanus Oil		*p* (n = 18) Placebo Group		*p*^a^ t_0_ vs. t_12_	*p*^a^ t_0_, t_12_, t_16_
		Mean	SD	Mean	SD		
Fasting Glucose [mmol/L]	0	6.1	0.03	6.0	0.34	0.437	0.717
	12	5.9 * ^b^	0.47	5.9	0.44		
	16	5.9 * ^b^	0.43	5.9	0.49		
Fasting Insulin [mU/L]	0	15.8	6.21	14.3	7.64	0.014	0.050
	12	12.9 * ^b^	5.80	16.5	13.66		
	16	14.0	7.06	14.4	10.74		
HOMA-Index	0	4.38	1.66	3.89	2.03	0.028	0.094
	12	3.48 * ^b^	1.54	4.57	3.97		
	16	3.83	1.94	3.94	3.05		
HbA_1c_ [%]	0	5.33	0.36	5.42	0.41	0.331	0.362
	12	5.41	0.28	5.61	0.30		
	16	5.48	0.23	5.58	0.37		
2 h plasma glucose (mmol/L)	0	6.6	1.26	5.9	0.98	0.964	0.948
	12	6.1	1.13	5.4	0.97		
	16	5.9	1.44	5.4	1.21		
AUC-Glucose_0–2h_	0	955	158	927	127	0.949	0.953
	12	917	203	892	143		
	16	876	176	862	133		
AUC-Insulin_0–2h_	0	8845	3572	8303	4070	0.837	0.841
	12	8900	3556	8493	3638		
	16	8926	3960	7687	3519		
HIRI ^†^	0	5.62	2.48	4.38	1.24	0.021	0.058
	12	4.56 * ^b^	2.44	4.60	1.59		
	16	5.06	2.55	4.06	1.38		
MISI ^‡^	0	0.158	0.12	0.174	0.13	0.748	0.927
	12	0.175	0.12	0.190	0.11		
	16	0.157	0.08	0.182	0.11		
CRP (mg/L)	0	2.81	3.71	3.88	2.54	0.728	0.478
	12	2.58	2.55	3.42	2.59		
	16	2.98	3.71	3.06	1.89		

Values are given as mean ± SD; HOMA-Index, homeostatic model of assessment-insulin resistance; HIRI, hepatic insulin resistance index; MISI, muscle insulin sensitivity index; CRP, C-reactive protein. *p* ^a^-values represent the time * intervention interaction analysed with a two-way repeated measure. *p* ^b^-values represented the time effect within the groups; * *p* < 0.05 after post hoc Bonferroni correction from t_0_ to t_12_ and t_0_ to t_16_, respectively; no significant differences were observed from t_12_ to t_16_. ^†^ HIRI is measured as 10^6^ [AUC Glucose (mg/min^−1^/dL) × AUC Insulin (mU/min^−1^/mL)] after standardized 5-point OGTT. ^‡^ MISI=dGlucose/dt÷Plasma Insulin [mean during OGTT mU/L].

**Table 4:** Omega-3 Fatty acid content [%] of total fatty acids in in red blood cells (RBC) at the beginning (t_0_), after 12 (t_12_) and 16 (t_16_) weeks of Calanus oil intervention.

Parameter	t	CO (n = 25) Calanus Oil		*p* (n = 18) Placebo Group		*p*^a^ t_0_ vs. t_12_	*p*^a^ t_0_, t_12_, t_16_
		Mean	SD	Mean	SD		
ALA (C18:3n-3)	0	0.15	0.04	0.17	0.05		
	12	0.16	0.06	0.17	0.06	0.389	0.216
	16	0.16	0.06	0.19	0.06		
SDA (C18:4n-3)	0	0.047	0.011	0.041	0.016	0.160	0.087
	12	0.054 * ^b^	0.013	0.043	0.012		
	16	0.054	0.011	0.050	0.015		
EPA (C20:5n-3)	0	0.99	0.33	1.23	0.62	<0.001	<0.001
	12	1.40 ** ^b^	0.44	1.05	0.48		
	16	1.39 ** ^b^	0.40	1.09	0.51		
DHA (C22:6n-3)	0	5.46	1.14	6.14	1.47	<0.001	<0.001
	12	6.31 ** ^b^	1.24	5.84	1.42		
	16	6.34 ** ^b^	1.17	5.84	1.51		
Omega-3 Index	0	6.45	1.37	7.37	1.96	<0.001	<0.001
	12	7.71 ** ^b^	0.54	6.89 ** ^b^	1.78		
	16	7.72 ** ^b^	1.43	6.94 ** ^b^	1.87		

Values are given as mean ± SD. ALA, alpha-linolenic acid; SDA, stearidonic acid; EPA, eicosapentaenoic acid; DHA, docosahexaenoic acid. *p* ^a^-values represent the time * intervention interaction analysed with a two-way repeated measure ANOVA. *p* ^b^-values represented the time effect within the groups; * *p* < 0.05, ** *p* < 0.001 after post hoc Bonferroni correction from t_0_ to t_12_ and t_0_ to t_16_, respectively; no significant differences were observed from t_12_ to t_16_.

## Data Availability

Several data, which were generated during the current study, are not publicly available, but are available from the corresponding author upon reasonable request.

## Appendix A

**Table A1:** Body composition at the beginning (t0), after 12 (t12) and 16 (t16) weeks of intervention.

Parameter	t	CO (n = 25) Calanus Oil		*p* (n = 18) Placebo Group		*p*^a^ t_0_ vs. t_12_	*p*^a^ t_0_, t_12_, t_16_
		Mean	SD	Mean	SD		
Weight (kg)	0	93.0	19.7	95.8	19.3	0.686	0.840
	12	92.8	18.9	95.6	20.2		
	16	92.8	19.1	95.6	20.6		
BMI (kg/m^2^)	0	30.9	5.21	32.9	5.32	0.761	0.951
	12	30.9	5.08	32.8	5.45		
	16	30.9	5.13	32.8	5.61		
Phase angle	0	5.25	0.56	5.21	0.47	0.389	0.385
	12	5.23	0.57	5.29	0.65		
	16	5.30	0.65	5.41	0.58		
LBM (kg)	0	58.9	15.03	59.8	14.60	0.193	0.313
	12	58.7	14.12	58.9	15.06		
	16	58.5	14.25	59.7	14.92		
BCM (kg)	0	28.4	7.91	28.6	7.19	0.436	0.168
	12	28.1	6.98	28.4	7.92		
	16	28.3	7.40	29.3	8.26		
FM (kg)	0	34.2	10.18	35.9	10.35	0.249	0.245
	12	34.1	9.76	36.7	10.01		
	16	34.3	9.82	35.9	10.72		
TBL (L)	0	43.1	10.99	43.8	10.68	0.102	0.355
	12	42.8	10.34	43.1	11.02		
	16	42.9	10.41	43.7	10.94		

Values are given as mean ± SD. BMI, body mass index; LBM, lean body mass; BMC, body cell mas; FM, fat mass; TBW, total body water. *p* ^a^-values represent the time intervention interaction analysed with a two-way repeated measure ANOVA.

**Table A2:** Daily dietary intake calculated from 3-day dietary records at the beginning (t0), after 12 (t12) and 16 (t16) weeks of intervention.

Parameter	t	CO (n = 25) Calanus Oil		*p* (n = 18) Placebo Group		*p*^a^ t_0_ vs. t_12_	*p*^a^ t_0_, t_12_, t_16_
		Mean	SD	Mean	SD		
Energy intake (kcal/d)	0	2091	373	2279	690	0.053	0.141
	12	2161	443	1943 * ^b^	542		
	16	2057	528	2127	650		
Protein (%E/d)	0	15.6	2.4	16.9	4.9	0.540	0.696
	12	15.6	2.6	17.8	5.9		
	16	16.2	3.0	16.9	5.2		
Fat (%E/d)	0	38.1	6.3	37.6	11.0	0.600	0.399
	12	37.2	6.7	39.5	6.9		
	16	40.7	7.6	38.7	9.3		
CHO (%/d)	0	40.8	6.2	39.2	9.4	0.806	0.510
	12	39.9	6.6	37.5	9.9		
	16	36.5	6.6	41.4	7.6		
Fibre (g/d)	0	22.4	6.4	25.5	10.4	0.313	0.407
	12	24.2	11.1	23.0	9.3		
	16	20.7	5.9	20.6	8.0		
SFA (g/d)	0	27.0	9.1	28.1	10.0	0.217	0.256
	12	24.1	8.5	19.4 * ^b^	8.6		
	16	23.8	9.0	25.2	13.2		
MUFA (g/d)	0	20.8	8.9	23.8	10.5	0.425	0.702
	12	18.2	6.8	18.4	7.6		
	16	18.9	7.5	18.2	9.6		
PUFA (g/d)	0	10.7	5.0	9.6	4.1	0.171	
	12	7.5	3.8	9.6	6.5		0.258
	16	8.4	4.2	8.5	4.6		
DHA (g/d)	0	0.29	0.36	0.32	0.35	0.373	
	12	0.16	0.23	0.29	0.55		0.706
	16	0.30	0.30	0.46	0.66		
EPA (g/d)	0	0.21	0.32	0.43	0.61	0.730	0.915
	12	0.09	0.16	0.17	0.33		
	16	0.18	0.25	0.39	0.53		

Values are given as mean ± SD. CHO, Carbohydrates; SFA, saturated fatty acids; MUFA, monounsaturated fatty acids; PUFA, polyunsaturated fatty acids; DHA, docosahexaenoic acid; EPA, eicosapentaenoic acid. *p* ^a^-values represent the time * intervention interaction analysed with a two-way repeated measure ANOVA. *p* ^b^-values represented the time effect within the groups. * *p* < 0.05 after post hoc Bonferroni correction from t_0_ to t_12_ and t_0_ to t_16_, respectively; no significant differences were observed from t_12_ to t_16_.
